# Risk factors for prolonged postoperative ICU stay in the patients with Stanford type A aortic dissection

**DOI:** 10.1186/s13019-024-02548-7

**Published:** 2024-02-03

**Authors:** Haiyuan Liu, Shuaipeng Zhang, Chengxin Zhang, Qinyun Gao, Yuyong Liu, Fangfang Liao, Shenglin Ge

**Affiliations:** 1 Department of Cardiovascular Surgery, First Affiliated Hospital of Anhui Medical University, Hefei, 230061 Anhui Province China; 2 Department of Infection Management, Second People’s Hospital of Hefei, Hefei, 230011 Anhui Province China

**Keywords:** Stanford type A aortic dissection, Prolonged ICU stay, Risk factors

## Abstract

**Objective:**

To investigate the independent risk factors for postoperative prolonged ICU stay in patients with Stanford type A aortic dissection (TAAD) and assess the clinical outcomes of prolonged ICU stay.

**Method:**

The clinical data of 100 patients with TAAD admitted to the Department of Cardiovascular Surgery, First Affiliated Hospital of Anhui Medical University from December 2018 to September 2022 were retrospectively collected and analyzed. Patients were divided into two groups, based on the postoperative ICU stay (7 days as the threshold), regular ICU stay group (< 7 days) and prolonged ICU stay group (≥ 7 days). First, preoperative and intraoperative materials were collected for univariate analysis. Then, the significant variables after univariate analysis were analyzed using logistic regression, and the final independent risk factors for prolonged ICU stay were determined. Meanwhile, the postoperative clinical outcomes were analyzed with the aim of assessing the clinical outcomes due to prolonged ICU stay.

**Results:**

There were 65 and 35 patients in the regular ICU stay group and the prolonged ICU stay group, respectively. In accordance with the result of univariate analysis in the two groups, emergency surgery (χ^2^ = 13.598; *P* < 0.001), preoperative urea nitrogen (t = 3.006; *P* = 0.004), cardiopulmonary bypass (CPB) time (t = 2.671; *P* = 0.001) and surgery time (t = 2.630; *P* = 0.010) were significant. All significant variates were analyzed through logistic regression, and it was found that emergency surgery (OR = 0.192; 95% CI: 0.065–0.561), preoperative urea nitrogen (OR = 0.775; 95% CI: 0.634–0.947) and cardiopulmonary time (OR = 0.988; 95% CI: 0.979–0.998) were independent risk factors for prolonged postoperative ICU stay. The Receiver Operating Characteristic (ROC) curves of these three factors were also effective in predicting postoperative prolonged ICU stay (Emergency surgery, AUC = 0.308, 95% CI: 0.201–0.415; Preoperative urea nitrogen, AUC = 0.288, 95% CI: 0.185–0.392; cardiopulmonary time, AUC = 0.340, 95% CI: 0.223–0.457). Moreover, compared with a single factor, the predictive value of combined factors was more significant (AUC = 0.810, 95% CI: 0.722–0.897). For the comparison of postoperative data in the two groups,, compared with the regular ICU stay group, the incidence of adverse events in the prolonged ICU stay group increased significantly, including limb disability of limbs (χ^2^ = 22.182; *P* < 0.001), severe organ injury (χ^2^ = 23.077; P < 0.001), tracheotomy (χ^2^ = 17.582; *P* < 0.001), reintubation (χ^2^ = 28.020; *P* < 0.001), 72 h tracheal extubation after surgery (χ^2^ = 29.335; *P* < 0.001), 12 h consciousness recovery after surgery (χ^2^ = 18.445; P < 0.001), ICU re-entering (χ^2^ = 9.496; *P* = 0.002) and irregular discharging (χ^2^ = 24.969; *P* < 0.001).

**Conclusion:**

Emergency surgery, preoperative urea nitrogen, and CPB time are risk factors for postoperative prolonged ICU stay after TAAD surgery. Furthermore, prolonged ICU stay is associated with worse clinical outcomes. Hence, a reasonable strategy should be adopted proactively focusing on the risk factors to shorten ICU stays and improve clinical outcomes.

TAAD is one of the most common critical diseases in cardiovascular surgery. Clinically, the outcomes of TAAD are adversely affected due to sudden severe onset, difficult treatment, and higher perioperative mortality. Currently, hypertension, dilated aortic aneurysm, and grafted aortic prosthesis-related medical factors are considered as the leading causes of TAAD, and the prevalence of TAAD is higher in the male population [1]. Undoubtedly, surgery is the preferred option for TAAD treatment; however, both diversity and uncertainty of postoperative outcomes are a concern because of advanced disease and different organ involvement by TAAD. With the dramatic updates for both surgical concepts and artificial prosthesis, the safety and effect of TAAD repair surgery is more reliable and worth trusting. Nevertheless, it should be emphasized that the importance of postoperative management is the same as surgical procedures or even larger, among which, as the critical transition stage from surgical trauma to rehabilitation, postoperative ICU management is the central axis, which is responsible for vital sign monitoring, medical intervention, and even timely salvage if necessary. In particular, prolonged ICU stay after surgery is associated with increased in-hospital mortality and morbidity and economic cost. It has been demonstrated that the length of ICU stay depends on a multifactorial mechanism [2]. For the assessment of ICU stay, EuroSCORE II, even though it is the newest risk predictive model of cardiac surgery, is poor in predicting the ICU stay without any assistance as the newest risk predictive model of cardiac surgery, is poor in predicting the ICU stay without any assistance from other models, which is less effective than APACHE IVa [3, 4]. Overall, an optimal and reasonable ICU management strategy is beneficial to outcome improvement and long-term survival; however, prolonged ICU stay due to unexpected clinical factors may reversely induce a series of adverse events, including ventilator-associated pneumonia, organ dysfunction, and delirium [5–7]. In an observational study of postoperative management, low preoperative hemoglobin level, prolonged cross-clamp times, decreased PaO_2_/FiO_2_ ratio, and changes in blood glucose within 1–4 h after surgery were associated with prolonged ICU stay [8]. The aim of this retrospective study was to explore the independent risk factors of postoperative prolonged ICU stay and their association with the outcomes of patients with TAAD.

## Subjects and the method

### Subjects

A total of 100 patients with TAAD admitted to the Department of Cardiovascular Surgery, the First Affiliated Hospital of Anhui Medical University, from December 2018 to September 2022 were enrolled as the subjects. Inclusion criteria were as follows: (1) TAAD was confirmed through imaging including enhanced CT angiography and/or echocardiography; (2) TAAD patients underwent the surgical procedures. Exclusion criteria were as follows: (1) There were no surgical indications due to advanced disease and/or subjective refusal from patients; (2) The clinical materials of patients were incomplete; (3) The patients were discharged irregularly within short ICU stay (< 7 days) due to varies unexpected reasons. Based on the literature and taking the clinical practice into consideration, all patients were divided into regular ICU stay group (< 7 days) and prolonged ICU stay group (≥ 7 days) [2, 9]. Comprehensively, the clinical criteria of ICU leave after surgery must be assessed as follows: (1) Total consciousness recovery and completed activities following physician’s instructions; (2)Mechanical ventilation weaning and the spontaneous breathing rate < 22 times/min; (3) The partial pressure of oxygen (PO_2_) and the partial pressure of carbon dioxide (PCO_2_) ≥ 80 mmHg and ≤ 50 mmHg, respectively; (4) Stable hemodynamics without severe and malignant arrhythmia; (5) The qualified postoperative heart function after assessment and supported by fewer vasoactive drugs.

### Method

Perioperative clinical materials of the patients were collected for retrospective analysis. Preoperative and intraoperative parameters included age, weight, laboratory tests, ejection fraction, surgery type, surgery time, CPB time, deep hypothermic circulatory arrest (DHCA) time, ultrafiltration, and cold blood reperfusion time (interval between DHCA and rewarming.). Postoperative materials included postoperative mental status, extubation time, limb disability, tracheotomy, and organ function injury. First, univariate analysis was performed for both preoperative and intraoperative materials, after which the significant variates were pooled and analyzed using logistic regression analysis to determine the final independent risk factors. ROC curves were also drawn to validate the specificity and sensitivity of prediction to prolonged ICU stay from independent risk factors. Finally, the comparison of postoperative materials in the two groups was performed to assess the effect of prolonged ICU stay on clinical outcomes.

### Statistics

Statistical software SPSS 26.0 was used for statistical analysis. The measurement data with normal distribution was expressed as mean ± standard deviation (χˉ ± s) and t test as well as χ^2^ test were used for intergroup comparison. *P* < 0.05 was considered statistically significant.

## Results

There were 71 male patients at age of 32–74 y (52.39 ± 8.73y) and 29 female patients at age of 26–76 y (55.24 ± 8.56y). For arterial cannulation sites, different cannulation sites were summarized as brachiocephalic trunk/innominate artery combined with femoral artery (53 patients), left common carotid artery combined with femoral artery (25 patients), axillary artery combined with femoral artery (12 patients), right subclavian artery combined with femoral artery (9 patients), and left subclavian artery combined with femoral artery (1 patient). For surgical procedures, different procedural types were summarized as aortic valve repair combined with ascending aorta replacement and Sun’s procedure (38 patients), Bentall combined with Sun’s procedure (24 patients), ascending aorta replacement combined with Sun’s procedure (19 patients), aortic valve repair combined with ascending aorta as well as right hemiarch replacement (6 patients), David combined with Sun’s procedure (6 patients), ascending aorta replacement combined with right hemiarch replacement (5 patients), David combined with right hemiarch replacement (1 patient), and abdominal aorta replacement combined with Sun’s procedure (1 patient). There were 65 patients in the postoperative regular ICU stay group and 35 patients in the prolonged ICU stay group.

### Univariate analysis of preoperative and intraoperative materials in the two groups

It was indicated that, through univariate analysis, the differences of emergency surgery (χ^2^ = 13.598; *P* < 0.001), preoperative urea nitrogen (t = 3.006; *P* = 0.004), CPB time (t = 2.671; *P* = 0.009) and surgery time (t = 2.630; P = 0.010) in the two groups were significant. There were no significant differences for the other variables (Table 1).

**Table 1:** Univariate analysis of preoperative and intraoperative materials in the two groups

Materials	Regular ICU stay (n = 65)	Prolonged ICU stay (n = 35)	t/χ^2^	*P*
Age (yrs)	53.17 ± 11.69	53.31 ± 10.11	0.062	0.951
Weight (kg)	71.00 ± 12.66	75.29 ± 12.72	1.612	0.110
Gender (Male/Female)	45/20	26/9	0.282	0.595
Hypertension	47(72.30%)	27(77.14%)	0.276	0.599
Diabetes mellitus	6(9.23%)	0(0.00%)	3.437	0.064
Cerebrovascular disease	5(7.69%)	6(17.14%)	2.075	0.150
Respiratory tract disease	2(3.08%)	2(5.71%)	0.412	0.521
Liver and renal disease	22(33.85%)	9(25.14%)	0.703	0.402
Marfan syndrome	2(3.08%)	0(0.00%)	1.099	0.295
Thyroid disease	3(4.62%)	1(2.86%)	0.183	0.669
Surgery history	26(40.00%)	13(37.14%)	0.078	0.780
Smoking history	22(33.85%)	8(22.86%)	1.308	0.253
Alcohol history	16(24.62%)	5(14.29%)	1.463	0.226
Digestive disease	9(13.85%)	4(6.15%)	0.118	0.732
Preoperative pericardial effusion	27(41.53%)	15(42.86%)	0.016	0.899
Preoperative valve involvement	21(32.30%)	11(31.43%)	0.008	0.928
Preoperative arrhythmia	14(21.53%)	10(28.57%)	0.617	0.432
Preoperative HCT	34.51 ± 8.46%	35.14 ± 7.61%	0.370	0.712
Preoperative HB (g/L)	111.43 ± 26.02	117.14 ± 22.72	1.093	0.277
Preoperative GLU (mmol/L)	7.17 ± 2.44	7.60 ± 1.89	0.903	0.369
Preoperative BUN (mol/L)	6.71 ± 2.35	9.55 ± 5.31	3.006	0.004^*^
Preoperative EF	60.11 ± 3.08%	59.40 ± 3.06%	-1.099	0.275
Emergency surgery	27(42.54%)	29(82.86%)	13.598	< 0.001^*^
CPB time (min)	242.71 ± 48.26	271.80 ± 58.24	2.671	0.009^*^
Cross-clamp (min)	183.35 ± 52.76	203.60 ± 69.27	1.636	0.105
DHCA time(min)	32.75 ± 12.85	31.17 ± 13.88	-0.571	0.569
Cold blood reperfusion time (min)	24.52 ± 11.46	27.66 ± 13.77	1.214	0.228
Ultrafiltration (ml)	4715.38 ± 1586.19	4880.00 ± 1760.31	0.476	0.635
Surgery time (min)	518.62 ± 86.04	571.09 ± 110.29	2.630	0.010^*^
HCT at CPB weaning	23.35 ± 3.85%	22.60 ± 3.27%	-0.983	0.328
HB at CPB weaning (g/L)	80.05 ± 11.60	78.91 ± 11.46	-0.467	0.641

^*^P < 0.05

### Logistic regression analysis

The remaining significant factors through univariate analysis were set as the independent variables and ICU stay was set as the dependent variable (ICU stay < 7 days after surgery = 1; ICU stay ≥ 7 days after surgery = 0), the Logistic regression (stepwise, forward) was used to determine the final independent risk factors. emergency surgery (OR = 0.192; 95%CI: 0.065–0.561), preoperative urea nitrogen (OR = 0.013; 95%CI: 0.634–0.947) and CPB time (OR = 0.988; 95%CI: 0.979–0.998) were significant factors (Table 2).

**Table 2:** Logistic regression analysis

Variates	Standard error	Ward	OR	*P*	95% CI
Emergency surgery	0.548	9.098	0.192	0.003	0.065–0.561
Preoperative BUN	0.102	6.203	0.775	0.013	0.634–0.947
CPB time	0.005	6.102	0.988	0.014	0.979–0.998

### Specificity and sensitivity of prediction to prolonged ICU stay based on independent risk factors

**Fig. 1:**
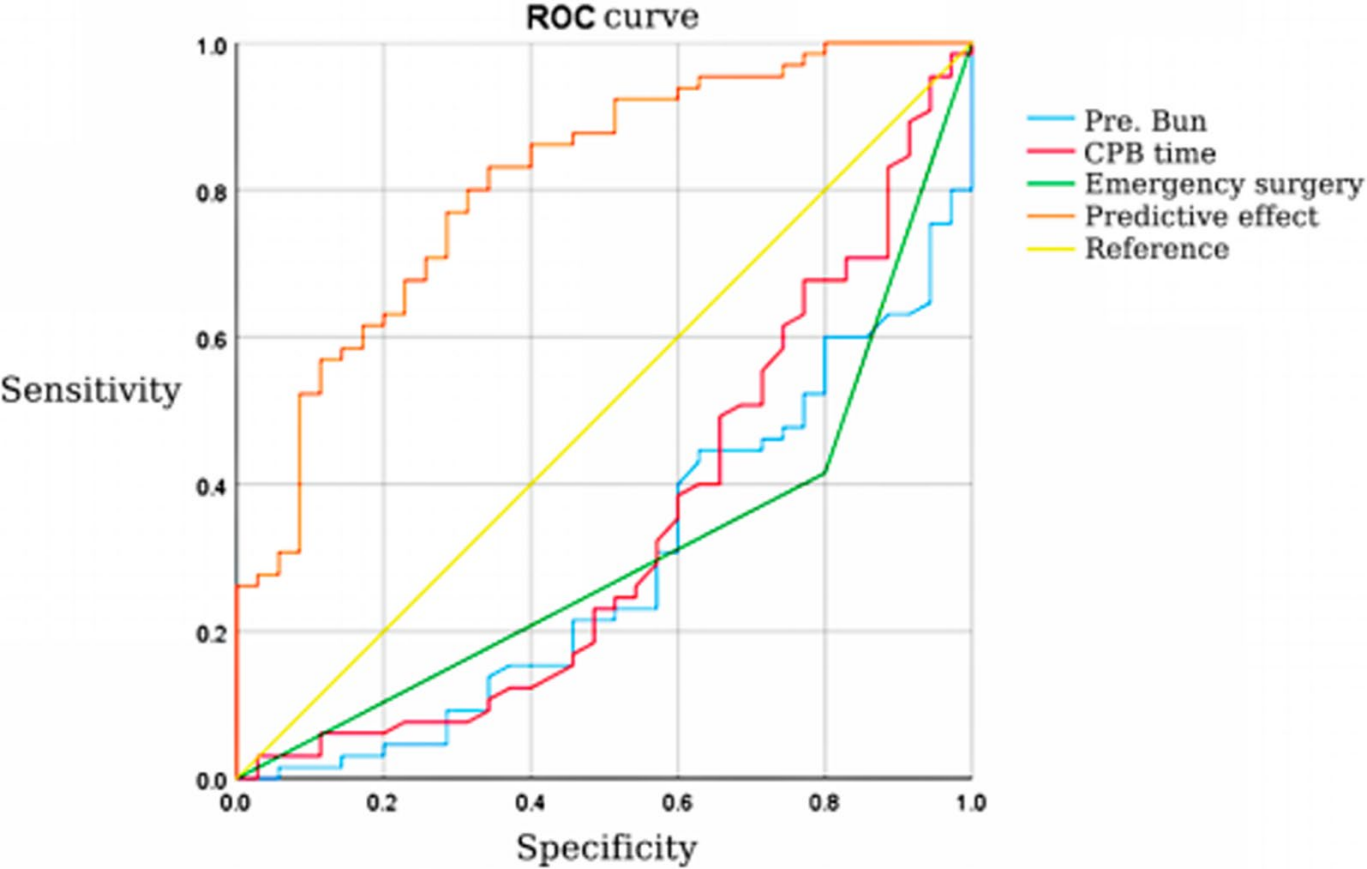
ROC curves for the prediction of prolonged ICU stay from independent risk factors

Based on the results of logistic regression analysis, emergency surgery, preoperative creatinine level, and CPB time were set as checking variables, and prolonged ICU stay was set as the state variable. A receiver operating characteristic (ROC) curve was drawn to validate the specificity and sensitivity of the predictive value of independent factors. The AUC of each checking variable and that of the combined factors were calculated. In accordance with the calculation of areas under the AUC curve for all factors, emergency surgery (AUC = 0.308; 95%CI: 0.201–0.415), preoperative urea nitrogen (AUC = 0.288; 95%CI: 0.185–0.392) and CPB time (AUC = 0.340; 95%CI: 0.223–0.457)were effective in predicting prolonged ICU stay; however, compared with any single factor, the predictive value of prolonged ICU stay with combined factors (AUC = 0.810; 95% CI: 0.722–0.897) increased significantly (Fig. 1 and Table 3).

**Table 3:** Specificity and Sensitivity of Prediction to Prolonged ICU stay based on Independent Risk Factors

Variates	The AUC	Standard error	*P*	95% C I
Emergency surgery	0.308	0.055	0.002	0.201–0.415
Preoperative BUN	0.288	0.053	0.001	0.185–0.392
CPB time	0.340	0.060	0.008	0.223–0.457
Combined factors	0.810	0.045	0.000	0.722–0.897

### Association between prolonged ICU stay and clinical outcomes

Compared with the regular ICU stay group, the incidence of adverse events in the prolonged ICU stay group increased significantly, including limb disability (χ^2^ = 22.182; *P* < 0.001), severe organ injury (χ^2^ = 23.077; *P* < 0.001), tracheotomy (χ^2^ = 17.582; *P* < 0.001), reintubation (χ^2^ = 28.020; *P* < 0.001), 72 h tracheal extubation after surgery (χ^2^ = 29.335; *P* < 0.001), 12 h consciousness recovery after surgery (χ^2^ = 18.445; *P* < 0.001), ICU re-entering (χ^2^ = 9.496; *P* = 0.002) and irregular discharging (χ^2^ = 24.969; *P* < 0.001). (Table 4).

**Table 4:** Comparison of postoperative clinical outcomes between the two groups

Material	Regular ICU stay (n = 65)	Prolonged ICU stay(n = 35)	χ^2^	*P*
Limb disability	0(0.00%)	12(34.29%)	22.182	< 0.001
Severe organ injury	2(3.08%)	14(40.00%)	23.077	< 0.001
Tracheotomy	0(0.00%)	10(15.38%)	17.582	< 0.001
Reintubation	2(3.08%)	16(45.71%)	28.020	< 0.001
72 h tracheal extubation after surgery	54(83.08%)	10(28.57%)	29.335	< 0.001
12 h consciousness recovery after surgery	56(86.15%)	16(45.71%)	18.445	< 0.001
ICU re-entering	4(6.15%)	10(28.57%)	9.496	0.002
Irregular discharging	3(4.62%)	16(45.71%)	24.969	< 0.001

## Discussion

Recently, the number of younger TAAD patients in China has been increasing gradually, and the risk incidences for both dissection rupture and dissection-related complications are relatively larger due to limited medical resources and undeveloped socioeconomic status. Therefore, regionally and actually, the postoperative clinical outcomes of TAAD vary [10]. Clinically, postoperative ICU management is an indispensable element for both physical and mental recovery after TAAD surgery, which is valuable in stabilizing circulation, respiration, coagulation, and organ functions [11].

In the current study, TAAD was selected as the targeted disease to explore how to optimize the ICU management strategy focusing on critical cardiovascular diseases. The results indicated that emergency surgery, preoperative urea nitrogen, and CPB time were associated with prolonged postoperative ICU stay. Meanwhile, compared with the regular ICU stay group, the incidence of adverse events in the prolonged ICU stay group, including limb disability, severe organ injury, tracheotomy, reintubation, 72 h tracheal extubation after surgery, 12 h consciousness recovery after surgery, ICU re-entering, and irregular discharge, was significantly higher.

In clinical practice, TAAD is commonly characterized by acute onset and progressive invasion of the arterial system. Thus, undoubtedly, emergency surgery is the preferred treatment for TAAD. Nevertheless, although effective for life-saving purposes, it is inadequate and difficult to handle several unexpected conditions during emergency surgery, such as organ malperfusion syndrome secondary to TAAD. Girdauskas et al. [12] found that preoperative malperfusion is the major factor associated with systemic inflammatory response, acute respiratory dysfunction, and renal failure, leading to prolonged ICU stay due to longer mechanical ventilation and dialysis. In a survival study conducted by Wolfe et al. [13], among patients with TAAD, more than 40% of whom were complicated with varying preoperative malperfusion, it was found that the highest odds of in-hospital mortality was from mesenteric malperfusion followed by cerebral malperfusion. Currently, rather than the priority of TAAD repair emergency surgery, perfusion improvement of malperfusion organs is the first intervention goal [14, 15]. In a study conducted by Yang et al. [16], for patients with TAAD complicated with mesenteric artery malperfusion, initial endovascular intervention therapy was activated to improve perfusion flow of the mesenteric artery following elective open surgical dissection repair. Collectively, it was found that compared with non-malperfusion patients, there was no significant difference in mortality for the surviving patients with malperfusion after endovascular intervention, although higher in-hospital mortality of 40% at admission. Compared with the “central repair-first” strategy, both in-hospital and 30-day mortality of patients undergoing “revascularization-first” strategy are significantly lower, and except for a longer hypothermic cardiac arrest period, no more adverse aortic events are found [17]. Furthermore, compared with TAAD patients with non-malperfusion, the average time of redo after emergency surgery for patients with malperfusion was shortened to 2 days [18]. Moreover, for patients complicated with preoperative cerebral malperfusion, the comprehensive feasibility of emergency surgery should be assessed. In a retrospective study, the hospital mortality of patients with cerebral malperfusion was significantly higher, especially for those presenting with coma, whereas axillary artery cannulation was considered as a protective factor [19]. Okita et al. [20] recommended that reperfusion of the carotid artery before TAAD repair surgery among patients with brain malperfusion secondary to TAAD is associated with fewer neurological adverse events. Thus, early established anatomic bypass of the left/right carotid artery for cerebral reperfusion maintenance is associated with a reduced incidence of postoperative neurological complications [21]. However, the association between age and emergency surgery remains controversial. First, for younger patients, the surgery strategy mainly depends on the involvement of aortic branches, whereas for older patients, the strategy mainly focuses on the structure and function of the left ventricle [22]. Older age (≥ 80y) is associated with higher intraoperative mortality compared with conservative treatment. Although there is considerable short-term outcome improvement, there is no significant difference in long-term outcome improvement for emergency surgery [23]. During the 5-year follow-up after surgery, no significant survival benefits were observed compared with conservative treatment, and a higher incidence of postoperative stroke was the leading factor of hospital mortality [24]. However, it has also been indicated that older age (> 70y) should not be considered as a factor determining emergency surgery, although it is accompanied by higher postoperative stroke rates, as indicated by a survey in the UK [25]. Therefore, the assessment of surgical indications for older patients with TAAD should be performed with caution.

Urea nitrogen is one of the most important biomarkers of renal function. Elevated urea nitrogen is commonly found in patients with impaired renal function. Arginine vasopressin is activated excessively to maintain adequate systemic perfusion, which is affected by both low cardiac output and myocardial ischemia due to coronary artery involvement by TAAD. Subsequently, the permeability of urea nitrogen into the collecting duct increases dramatically under the interaction of arginine vasopressin [26]. Liu et al. [27] demonstrated that urea nitrogen at admission (6.95 mmol/L) is valuable in predicting in-hospital mortality with sensitivity and specificity of 78.9% and 72.2%, respectively. In our study, it was found that elevated preoperative urea nitrogen is a risk factor for prolonged postoperative ICU stay, which suggests that preoperative renal function plays a critical role in affecting clinical outcomes. This may partly explain why renal arteries are involved in TAAD, causing renal malperfusion before surgery and leading to secondary impact on renal function. Compared with patients without renal dysfunction before surgery, CPB time, cross-clamp time, and cardiac arrest time are significantly longer in patients with renal malperfusion. Meanwhile, preoperative renal malperfusion is also associated with postoperative acute renal injury, and temporary or even permanent hemodialysis therapy is necessary as an alternative treatment for renal function; to some extent, ICU stay may be prolonged [28]. Similarly, another biomarker of renal function, creatinine > 1.5 mg/dL, is also correlated with the incidence of postoperative adverse events, including mortality [29]. Furthermore, Wu et al. [30] found that compared with patients with higher 30-day mortality after TAAD surgery, the pre-operative creatinine levels among those with lower mortality decreased significantly (9.61 vs 13.41 mol/dL). Focusing on the classification of preoperative renal function for patients with TAAD, it has been suggested that moderate to severe preoperative renal dysfunction is an independent risk factor for postoperative hemodialysis, which probably affects the regular ICU stay [31]. Moreover, Cai et al. [7]indicated that there is a positive association between renal dysfunction and postoperative delirium, potentially causing prolonged postoperative ICU stay.

CPB, which is responsible for stable vital signs and a safe surgical platform during cardiac arrest, is essential during TAAD repair surgery. However, because of its exogenous nature, the immune system is activated to induce an inflammatory response when the physiological circulation is maintained and supported through the established artificial circuit of CPB. Potentially and sequentially, organ injury or dysfunction, internal environment disturbance, physiological regulation inactivation of the secretion system, and time-dependent mechanical damage of blood components are gradually observed, as expected. In a study concentrating on the association between CPB time and postoperative renal function changes conducted by Xu et al. [32], the incidence of postoperative acute renal injury increased by 17% with every additional 10 min of CPB time. Persistent progressive injury of the renal tubule during dramatic longer CPB is a reasonable and reliable explanation for postoperative renal dysfunction [33]. In a study of neurological outcomes, CPB time ≥ 300 min is confirmed as a risk factor for postoperative stroke [34]. Among patients with postoperative infection, gram-negative bacteria are the major pathogen within the earlier bloodborne infection phase after surgery because of the possibility of Enterobacter migration induced by CPB [35]. During the surgical procedure of aortic arch replacement combined with elephant trunk stent implantation, there was a significant association between CPB time and worse outcome; for every additional 10 min, the possibility of worse outcome increases by 10% [36]. Based on the findings from an observational study of medium- and long-term outcomes conducted by Zheng et al. [37], there was a correlation between CPB time and 90-day mortality after surgery (every added 10 min, the mortality increased by 16%). The results of our study are similar to the conclusions mentioned above. A longer CPB time is associated with an increased incidence of adverse postoperative events, which in turn leads to prolonged ICU stay for systemic rehabilitation and organ function improvement. Note that DHCA is a critical stage during the CPB period for TAAD repair surgery with the aim of providing a relatively bloodless surgical field for arch replacement. However, the systematic impact of DHCA should not be ignored under extreme deep hypothermia, ischemia, and hypoxemia. It has been demonstrated that DHCA > 40 min is a significant risk factor for postoperative neurological complications [38]. Therefore, although no significant difference was found in DHCA time between the two groups in the current study, it is proposed that DHCA should be controlled as strictly as possible to alleviate ischemic and hypoxic related injury.

Currently, regular antegrade cerebral perfusion is thought to be an effective strategy of brain protection that can support and maintain suitable cerebral perfusion to extend DHCA time accordingly [39]. Later, in accordance with the specificity and sensitivity of ROC, it has been shown that the combination of emergency surgery, preoperative urea nitrogen, and CPB time is more effective in predicting prolonged ICU stay, which means that the interaction from multiple factors more likely induces prolonged ICU stay. Therefore, multidimensional and comprehensive thinking should be established and maintained in clinical diagnosis and treatment.

This study also has some limitations. First, the relatively smaller sample in this study may have led to insufficient statistical efficiency. Second, as a single-center study, the collected data in our study might be less comprehensive and validated to a broader patient population. Moreover, the association between both surgery types and arterial cannulation sites, which might also be associated with the outcomes, was not included in our analysis. Therefore, more multi-center studies are needed for more comprehensive clinical exploration and validation and long-term follow-up observation. In conclusion, emergency surgery, preoperative urea nitrogen, and CPB time are independent risk factors for prolonged postoperative ICU stay, and the combination of these three factors is more reliable in predicting ICU stay. In addition, the incidence of adverse events after TAAD surgery is significantly higher among patients with a prolonged postoperative ICU stay. In the future, for TAAD patients, especially those complicated with malperfusion, indications for emergency surgery, effective protection of renal function before surgery, and real-time optimization of CPB strategy are meaningful for improving postoperative ICU management.

## Abbreviations

HCT: Haematocrit
HB: Haemoglobin
GLU: Glucose
BUN: Urea nitrogen
EF: Ejection fraction
CPB: Cardiopulmonary bypass
DHCA: Deep hypothermia cardiac arrest
CABG: Coronary artery bypass graft
CI: Confidence interval
AUC: Area under the curve
